# Correlation of *BRCA1*, *TXR1* and *TSP1* mRNA expression with treatment outcome to docetaxel-based first-line chemotherapy in patients with advanced/metastatic non-small-cell lung cancer

**DOI:** 10.1038/sj.bjc.6606027

**Published:** 2010-12-14

**Authors:** C Papadaki, E Tsaroucha, L Kaklamanis, E Lagoudaki, M Trypaki, K Tryfonidis, D Mavroudis, E Stathopoulos, V Georgoulias, J Souglakos

**Affiliations:** 1 Laboratory of Tumor Cell Biology, School of Medicine, University of Crete, Heraklion, Crete, Greece; 2 8th Department of Pulmonary Diseases, ‘Sotiria’ General Hospital, Athens, Greece; 3 Department of Pathology, Onassis Center for Cardiovascular Diseases, Athens, Greece; 4 Department of Pathology, University General Hospital of Heraklion, Crete, Greece; 5 Department of Medical Oncology, University General Hospital of Heraklion, Voutes and Stavrakia, PO BOX 1352, Heraklion, Crete, Greece

**Keywords:** *BRCA1*, NSCLC, taxanes, *TSP1*, *TXR1*

## Abstract

**Background:**

We explored the predictive significance of *BRCA1*, *TXR1* and *TSP1* expression in non-small-cell lung cancer (NSCLC) patients treated with docetaxel in association with cisplatin or gemcitabine.

**Methods:**

To analyse *BRCA1, TXR1* and *TSP1* mRNA expression from microdissected primary tumours of 131 patients with stage IIIB (wet) and IV NSCLC, RT–qPCR was used.

**Results:**

The mRNA levels of *TXR1/TSP1* were inversely correlated (Spearman's test: −0.37; *P*=0.001). Low *TXR1* mRNA levels were associated with higher response rate (RR; *P*=0.018), longer median progression-free survival (PFS; *P*=0.029) and median overall survival (mOS *P*=0.003), whereas high *TSP1* expression was correlated with higher RR (*P*=0.035), longer PFS (*P*<0.001) and mOS (*P*<0.001). Higher *BRCA1* mRNA expression was associated with higher RR (*P*=0.028) and increased PFS (*P*=0.021), but not mOS (*P*=0.4). Multivariate analysis demonstrated that low *TXR1*/high *TSP1* expression was an independent factor for increased PFS (HR 0.49; 95% CI 0.32–0.76; *P*<0.001) and mOS (HR 0.37; 95% CI 0.2–0.58; *P*<0.001), whereas high *BRCA1* expression was correlated with increased PFS (HR 0.53; 95% CI 0.37–0.78; *P*=0.001).

**Conclusions:**

These data indicate that *TXR1/TSP1* and *BRCA1* expression could be used for the prediction of taxanes' resistance in the treatment of NSCLC.

Non-small-cell lung cancer (NSCLC) is the most common visceral malignancy worldwide for both sexes, accounting for ∼1.0 million cancer deaths per year (Jemal *et al*, 2009). Several drugs are used for disease control but the combinations of cisplatin with taxanes, vinorelbine and gemcitabine, have been established as the new standards of care. Treatment of patients with advanced/metastatic NSCLC with regimens combining platinum compounds and taxanes have extended the median survival time to 8–11 months and the 1-year survival rate to 30–40% (Georgoulias *et al*, 2001; Schiller *et al*, 2002).

There is a growing body of evidence regarding the genetic factors that could predict response to chemotherapy in NSCLC. The *BRCA1* has emerged as one of the most appealing genetic markers for the customisation of chemotherapy in NSCLC. It has multiple roles not only in DNA damage repair but also in cell cycle regulation, transcriptional control, ubiquitination and apoptosis (Georgoulias *et al*, 2001; Mullan *et al*, 2001; Kennedy *et al*, 2002), and it may be a regulator of mitotic spindle assembly as it colocalises to the microtubules of the mitotic spindle and to the centromeres (Lotti *et al*, 2002). Decreased *BRCA1* mRNA expression in a breast cancer cell line led to a greater sensitivity to cisplatin and etoposide and to a greater resistance to the microtubule-interfering agents paclitaxel and vincristine (Lafarge *et al*, 2001). Reconstitution of wild-type *BRCA1* into *BRCA1*-negative HCC1937 breast cancer cells resulted in a 20-fold increase in cisplatin resistance and, in contrast, a 1000- to 10 000-fold increase in sensitivity to paclitaxel and vinorelbine (Quinn *et al*, 2003). This differential modulating effect of *BRCA1* mRNA expression was also observed in tumour cells isolated from malignant effusions of NSCLC or gastric cancer patients (Wang *et al*, 2008), as well as in patients with ovarian cancer (Quinn *et al*, 2007), where high *BRCA1* mRNA levels correlated negatively with cisplatin sensitivity and positively with docetaxel sensitivity. The value of the *BRCA1* as a predictive marker for the treatment customisation in NSCLC has, also, been investigated in several retrospective studies (Taron *et al*, 2004; Boukovinas *et al*, 2008) and in one prospective trial (Boukovinas *et al*, 2008; Rosell *et al*, 2009).

Another mechanism of taxane resistance, which has been recently described, suggests that overexpression of a previously unknown gene, the *taxol resistance gene 1* (*TXR1)* or proline rich 13 (*PRR13*), prevents apoptosis in a human prostate cancer cell line (Lih *et al*, 2006). This effect was mediated by the downregulation of the antiangiogenic and proapoptotic glycoprotein *thrombospondin 1* (*TSP1*). Moreover, the sensitivity of cells in taxanes was increased by either inactivation of *TXR1* using siRNA or by activating signalling through the integrin-associated protein (CD47 receptor) (Lih *et al*, 2006). A retrospective study conducted, from our group in 96 adenocarcinoma NSCLC patients treated with docetaxel–gemcitabine, provided evidence for *in vivo* relevance of this model. Our results confirmed the *in vitro* evidence that overexpression of *TXR1* was significantly correlated with downregulation of *TSP1* expression (*P*<0.0001), and the expression of both genes was significantly correlated with treatment outcome (Papadaki *et al*, 2009).

Based on these data we decided to conduct a retrospective study in order to investigate the predictive significance of *BRCA1* and *TXR1–TSP1* mRNA expression in NSCLC patients treated with docetaxel-based doublets. The main goal of the study was to validate, in an independent patients' cohort, the predictive significance of *TXR1–TSP1* expression in different histologies of NSCLC and across with chemotherapy regimens combining docetaxel with platinum compounds (DC) or gemcitabine (DG).

## Patients and methods

### Patients

A total of 131 consecutive patients with histologically confirmed stage IIIB (with pleural effusion) and IV NSCLC and available tumour material for molecular analysis, who were treated with docetaxel*–*gemcitabine or docetaxel*–*cisplatin regimens as first-line treatment, at the University Hospital of Heraklion (Crete, Greece) between January 2003 and December 2007 were enroled. The above group of patients represents an independent patients' cohort (consecutive patients, not overlapping cases with those of the previous report) and was used as a confirmatory group (patients with different histologies and different taxanes regimens) in order to validate the results reported previously (Boukovinas *et al*, 2008; Papadaki *et al*, 2009). The study has been approved by the institutional ethics committee and all patients gave their informed consent for the use of the tissue material for translational research.

### Specimens' characteristics and assay methods

In order to ensure the validity of the specimen and select the most appropriate area for microdissection, all paraffin-embedded tumours were reviewed by two independent pathologists (EL and ES). Serial sections of 5 *μ*m thickness were prepared and then stained with nuclear Fast Red (Sigma-Aldrich, St Louis, MO, USA). Cancer cells were procured using an Eppendorf piezoelectric microdissector (Eppendorf, Hamburg, Germany) (Harsch *et al*, 2001).

The pellet of microdissected cells was subsequently submitted for RNA extraction with Trizol LS (Invitrogen, Carlsbad, CA, USA), and the SuperScript III Reverse Transcriptase (Invitrogen) was used to prepare cDNA from 50 ng of total RNA for each gene as previously described (Papadaki *et al*, 2009). The quality of the extracted RNA was evaluated with amplification of *β*-actin before RT–qPCR. Only samples with cycle quantification (Cq) <30 were considered suitable for further analysis based on the validation experiments for the performance of the set of primers and probes (Supplementary Figure 1a and b). The primers and probe sets were designed using Primer Express 2.0 Software (AB, Foster City, CA, USA). All primers and probes sequence were previously reported (Boukovinas *et al*, 2008; Papadaki *et al*, 2009). Relative cDNA quantification for *BRCA1*, *TXR1*, *TSP1* and *β-actin* as an internal reference gene was done using the ABI Prism 7900HT Sequence Detection System (AB) (Supplementary Figures 2a–d).

Relative gene expression quantification was performed according to the comparative Ct method using *β*-actin as an endogenous control and commercial RNA controls (mRNA from lung and liver; Stratagene, La Jolla, CA, USA) as calibrators. In addition, RNA extracted from FFPE lung tissue of a normal individual (who was operated after an accident) was used as internal standard during the whole experiment. Final results were determined as follows: 2^−(ΔCt sample−ΔCt calibrator)^, where ΔC_T_ values of the calibrator and sample were determined by subtracting the C_T_ value of the target gene from the value of the reference gene. In all experiments, only triplicates with s.d. of the Ct value <0.25 were accepted, according to the manufacturer suggestions (AB 7900 and SDS 2.3 User guide; AB). In addition, genomic DNA contamination of each sample has been excluded by non-reverse transcription of RNA.

### Study design and statistics

This study was a retrospective analysis aiming to explore the predictive significance of *BRCA1, TXR1* and *TSP1* mRNA expression in patients with NSCLC treated with front-line DG or DC regimen, in an independent, confirmatory group of patients. All appropriate specimens of the primary tumour with >100 cells per section were included in the analysis. Objective responses were recorded according to the RECIST criteria (Therasse *et al*, 2000). All efficacy results were assessed on an intention-to-treat basis. Median progression-free survival (PFS) was measured from the date of first-line therapy initiation to the first radiographic documentation of disease progression or death and median overall survival (mOS) was calculated from the date of diagnosis of metastatic disease to death from any cause. Quantitative PCR analyses yielded values that were expressed as ratios between two absolute measurements (gene of interest: internal reference gene). Cutoff points were calculated according to the median value for the mRNA expression of each gene (Rosell *et al*, 2003; Taron *et al*, 2004). Samples with mRNA expression above or equal to the median were considered as samples with high expression, whereas those with value below the median as samples with low expression. All laboratory analyses were performed blinded to the clinical data.

Associations between treatment response and mRNA expression or baseline characteristics were assessed using Fisher's exact test for dichotomous variables or logistic regression for continuous variables. Kaplan–Meier curves were used to describe the proportion of subjects who remained free of events over the follow-up period. Associations between prognostic factors and PFS or OS were examined using Cox proportional hazards regression models; we report hazard ratio (HR) estimates and their 95% confidence intervals (CIs).

Cox regression models with interaction terms were used to assess whether mRNA expression effects varied across treatment subgroups. For each gene mRNA expression, two hypotheses were tested: (1) whether effect on first-line PFS and mOS varied according to regimen (DG or DC); and (2) whether the effect on first-line PFS and mOS varied according to tumour histology (squamous or non-squamous). All reported *P*-values were two sided and not adjusted for multiple testing.

## Results

### Patients' characteristics

Clinical data and representative samples from the primary tumours were collected from 131 consecutive patients treated with docetaxel-containing doublets in our centre. Successful amplification of both genes was achieved in all 131 specimens. Patient characteristics were all typical for NSCLC and are summarised in Table 1a.

In an intention-to-treat analysis, complete response (CR) was observed in 2 (2%) and partial response (PR) in 38 (28%) patients (overall response rate (RR) 30% 95% CI 24.3–39.2%). After a median follow-up period of 9.7 months (range 1.3–84.5), the median PFS was 4.2 months (95% CI 2.7–5.7) and the median OS was 11.1 months (95% CI 9.7–14.6).

### Genes' mRNA expression levels and response to treatment

The median mRNA expression levels were 4.28 (range 0.86–42.45) for *BRCA1*, 1.21 (range 0.02–7.8) for *TXR1* and 0.24 (range 0.02–1.87) for *TSP1*. There was no correlation between age, gender, performance status or stage of disease and *BRCA1*, *TXR1* or *TSP1* mRNA levels (all *P-*values >0.05). In comparison with non-squamous tumours (median 3.62; *P*=0.001), *BRCA1* was significantly higher in squamous tumours (median 8.1),whereas *TXR1* and *TSP1* expression was almost identical between the two histology groups (Table 1b) and in the same range with those reported for the previous patients' cohort (Papadaki *et al*, 2009). Furthermore, we observed the same inverse correlation between *TXR1* and *TSP1* mRNA expression (Spearman's test −0.37; *P*=0.001). For the *BRCA1,* as the expression values were significantly higher in squamous cell carcinomas in comparison with the adenocarcinomas (*P*=0.001), the cutoff values were calculated according to median expression levels in each group of squamous and non-squamous histology. Using these cutoff values, low (below the median) tumoural *BRCA1* expression was observed in 28 (50%) squamous and 38 (50%) non-squamous specimens, whereas high (above or equal to the median) in 28 (50%) and 37 (50%) patients with squamous and non-squamous tumours, respectively.

### *BRCA1*, *TXR1*, and *TSP1* expression levels and treatment outcome

**Figure 1:**
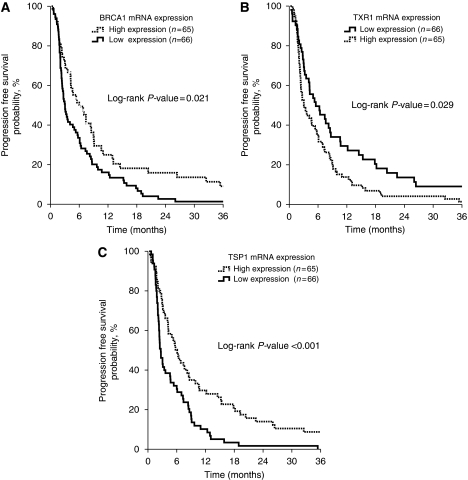
Progression-free survival (PFS) according to *BRCA1* (**A**), *TXR1* (**B**) and *TSP1* (**C**) mRNA expression.

**Figure 2:**
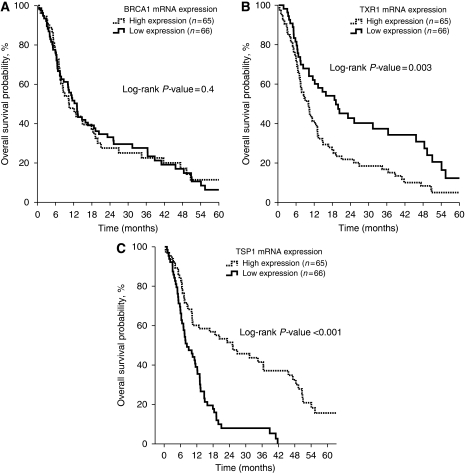
Median OS according to *BRCA1* (**A**), *TXR1* (**B**) and *TSP1* (**C**) mRNA expression.

Table 2 summarises the treatment outcomes according to *BRCA1*, *TXR1* and *TSP1* mRNA expression. Patients with high *BRCA1* mRNA expression had increased PFS (6.0 *vs* 3.0 months; *P*=0.021; Figure 1A) and RR (42 *vs* 20% *P*=0.028) in comparison with those with low *BRCA1* mRNA levels. Conversely, there was no difference in terms of OS according to the *BRCA1* mRNA expression (10.5 *vs* 11.2 months; *P*=0.4; Figure 2A). Patients with low *TXR1* expression experienced a longer PFS (5.5 *vs* 3.0 months; *P*=0.029; Figure 1B), OS (19.1 *vs* 10.0 months; *P*=0.003; Figure 2B) and RR (45 *vs* 18%, *P*=0.018) when compared with patients whose tumours had high *TXR1* mRNA expression. In addition, patients with high *TSP1* expression presented longer PFS (6.1 *vs* 2.6 months; *P*<0.001; Figure 1C), OS (25.1 *vs* 8.4 months; *P*<0.001; Figure 2C) and RR (41 *vs* 22% *P*=0.035) when compared with patients with low *TSP1* mRNA expression.

### Genes' mRNA expression and treatment outcome according to histological subtype and first-line regimen used

The correlation between high *BRCA1* mRNA expression and increased PFS was significant for both patients with squamous and non-squamous histology (interaction test *P*=0.61), whereas no significant correlation with mOS was found for either histological subtype (interaction test *P*=0.97; Table 3A). Similarly, the correlation between high *TXR1* mRNA expression and decreased PFS (interaction test *P*=0.21) and mOS (interaction test *P*=0.19) was comparable (Table 3A). Finally, high *TSP1* mRNA expression retained its predictive significance for increased PFS (interaction test *P*=0.34) and mOS (interaction test *P*=1.0) among patients with squamous and non-squamous histology (Table 3A).

Likewise, the correlation between high *TXR1* mRNA expression and decreased PFS (interaction test *P*=0.56) and mOS (interaction test *P*=0.38) was observed for patients receiving either DG or DC regimens (Table 3B). Moreover, PFS (interaction test *P*=0.48) and mOS (interaction test *P*=0.93) were significantly correlated with *TSP1* mRNA expression in either DG or DC treatment regimens (Table 3B). In contrast, high *BRCA1* mRNA expression was significantly correlated with increased PFS only in patients treated with DG regimen but not in those treated with DC (interaction test *P*=0.006); on the contrary, no such significant association with mOS was observed in either treatment group (interaction test *P*=0.61; Table 3B). In addition, 42 (63%) patients treated with first-line DG received second-line treatment with a cisplatin-based combination; *BRCA1* mRNA expression was significantly correlated with PFS (5.7 *vs* 2.2 months for patients with low and high mRNA expression; *P*=0.01) in this subgroup of patients.

### Univariate and multivariate analyses

Univariate analysis demonstrated that high *TXR1* (*P*=0.002) mRNA expression and stage IV (*P*=0.02) as diagnosis factors were significantly associated with decreased PFS, whereas high *TSP1* (*P*<0.001) and *BRCA1* (*P*=0.02) mRNA expression were associated with increased PFS. In addition, performance status of 2 (*P*=0.04), high *TXR1* (*P*=0.003) and low *TSP1* (*P*<0.001) mRNA expression were significantly associated with decreased OS (Table 4A). Cox proportional hazard analysis revealed that the combined expression of *TXR1* and *TSP1* (low *TXR1*/high *TSP1* expression) (HR 0.49; 95% CI 0.32–0.76; *P*<0.001) and *BRCA1* (HR 0.53; 95% CI 0.37–0.78; *P*=0.001) expression emerged as independent factors associated with increased PFS (Table 4B). Moreover, performance status (2 *vs* 0–1; HR 1.92; 95% CI 1.02–3.60; *P*=0.04) and *TXR1* and *TSP1* (low *TXR1*/high *TSP1* expression; HR 0.37; 95% CI 0.23–0.58; *P*<0.001) were independent prognostic factors for OS (Table 4b).

## Discussion

Downregulation of *TSP1* through *TXR1* overexpression has been proposed as a novel mechanism that modulates the cellular cytotoxicity of taxanes *in vitro* (Lih *et al*, 2006); indeed, in a previous report we have confirmed the clinical relevance of this mechanism in a group of patients with lung adenocarcinomas treated with a chemotherapy regimen combining docetaxel and gemcitabine (Papadaki *et al*, 2009). In this we evaluated the predictive significance of TXR1 overexpression/*TSP1* downregulation, together with *BRCA1* mRNA expression, in samples from patients with all histologies of NSCLC treated with either docetaxel/gemcitabine or docetaxel/cisplatin combinations.

We observed that the median expression values of *TXR1* and *TSP1* were almost identical between squamous and non-squamous carcinomas (*P-*value=0.92 and 1.0, respectively) and we confirmed that overexpression of *TXR1* was significantly correlated with downregulation of *TSP1* expression (*P*=0.001). In addition, multivariate analysis revealed that the favourable genotype (low *TXR1*/high *TSP1* expression) was an independent prognostic factor for increased PFS and survival, and was associated with a 51 and 63% reduction of the risk for progression or death, respectively. It is interesting to note that the predictive value of *TXR1* and *TSP1* expression for PFS and median OS was retained irrespectively of the tumour histology. All these results are in agreement with the published *in vitro* and *in vivo* data regarding the role of *TXR1–TSP1* expression in taxanes' resistance (Lih *et al*, 2006; Papadaki *et al*, 2009).

In contrast, median *BRCA1* expression was significantly different in squamous and non-squamous tumours (*P*=0.001), as has been previously reported (Rosell *et al*, 2007). Overexpression of *BRCA1* was significantly correlated with higher RR and PFS but not with mOS. The predictive value of *BRCA1* in PFS remained significant in both squamous and non-squamous groups (interaction test *P*=0.61). On the contrary, overexpression of *BRCA1* was significantly correlated with increased PFS in patients treated with DG but not in those treated with DC regimen (interaction test *P*=0.006). These results are in agreement with the current evidence for the differential predictive value of *BRCA1*, as low *BRCA1* expression confers increased sensitivity to cisplatin (Husain *et al*, 1998; Lafarge *et al*, 2001; Quinn *et al*, 2003, 2007) and resistance to antimicrotubule drugs such as docetaxel (Quinn *et al*, 2007), whereas high BRCA1 expression leads to resistance to cisplatin (Husain *et al*, 1998; Lafarge *et al*, 2001; Taron *et al*, 2004; Rosell *et al*, 2009) and sensitivity to docetaxel (Quinn *et al*, 2007; Boukovinas *et al*, 2008; Rosell *et al*, 2009). In addition, *BRCA1* overexpression is significantly correlated with those of *ERCC1* and *RRM1* in several studies (Rosell *et al*, 2007; Boukovinas *et al*, 2008; Wang *et al*, 2008; Bartolucci *et al*, 2009), providing another explanation for the lack of association of BRCA1 with OS.

High *BRCA1* mRNA expression has been associated with increased risk of relapse in patients with early (stage IB–IIB) NSCLC (Bartolucci *et al*, 2009), whereas BRCA1 haplotype could predict the outcome of NSCLC cancer patients treated with platinum-based chemotherapy, especially of those with squamous cell histology (Kim *et al*, 2008); these findings could explain the lack of significant association between BRCA1 expression and efficacy of the DC regimen observed in this study. Similarly, BRCA1 protein expression was not a predictive factor for response to treatment in patient with operable NSCLC treated with DC in the neoadjuvant setting (Kang *et al*, 2010). Finally, the poor correlation of *BRCA1* with OS, especially in patients treated with DG, could be partially explained by the fact that second-line cisplatin chemotherapy is more effective in patients with low BRCA1 expression as has been previously reported (Boukovinas *et al*, 2008), as was the case in the present study.

The effect of *TXR1*–*TSP1* mRNA expression on taxanes' cytotoxicity is independent of MDR phenotype (the ability of tumour cells to efflux taxanes through the upregulation of the ATP-dependent cell membrane glycoproteins) (Gottesman and Ling, 2006), as the overexpression of *TXR1* did not affect the cellular accumulation of [^3^H]-labelled-paclitaxel in the resistant cells and did not reduce the sensitivity to other agents that are also expelled from tumour cells by the MDR (Lih *et al*, 2006). Also, it seems to be independent from tubulin formation (the mutations of the *β-tubulin* gene that may interfere with the taxane-binding sites to microtubules) (Giannakakou *et al*, 1997, 2000a, 2000b; Kavallaris *et al*, 1997), as quantitative biochemical analysis of cell lines resistant to taxanes has shown the same tubulin dynamics as that of the parental (sensitive) cells and no increase in microtubules' isoforms that are commonly upregulated in tubulin-related resistance to taxanes (Gottesman and Ling, 2006; Lih *et al*, 2006).

Owing to the lack of a non-taxane-treated control group in our study, we cannot confirm that the effect of *TSP1* expression was taxane specific and not a simple predictive marker for response to chemotherapy. In many human cancers, *TSP1* expression is inversely correlated with progression, and it was also found to be an independent prognostic indicator (Neal *et al*, 2006; Guerrero *et al*, 2008). However, using cDNA microarrays, it has been previously shown that the induction of *TSP1* in docetaxel-treated head and neck squamous cell carcinoma cell lines increased cytotoxicity (Yoo *et al*, 2002). The TSP1 is considered a proapoptotic protein through the activation of CD47 cell surface receptor (Lih *et al*, 2006), which has previously been shown to result in not only caspase-independent (Mateo *et al*, 1999) but also caspase-dependent apoptosis (Manna *et al*, 2005). In addition, TSP1 is a strong inhibitor of angiogenesis and decreased levels of circulating TSP1 in certain inbred mouse strains are correlated with increased circulating endothelial precursors and susceptibility to cancers (Shaked *et al*, 2005). In NSCLC, reduced protein expression of TSP1 has been associated with increased microvessels count and unfavourable prognosis (Yamaguchi *et al*, 2002). On the other hand, overexpression of TSP1 has also been associated with aggressiveness and increased angiogenesis in lung cancer (Ioachim *et al*, 2006).

Despite the fact that the results of this study should be interpreted with caution because of the retrospective nature of the study, it seems that the *TXR1–TSP1* mRNA expression could be used as predictive markers for patients with NSCLC treated with docetaxel-based chemotherapy. The next step should be the evaluation of the significance of *TXR1/TSP1* expression in taxanes' chemosensitivity in other tumour types such as breast and ovarian cancer, where taxanes are commonly used in the daily clinical practice. If this will be the case, the clinical relevance of the *TXR1/TSP1* expression should be further validated in prospective adequately designed clinical trials.

## Figures and Tables

**Table 1a:** Patients' characteristics

	**Patients treated with DG or DC**	
	**Number**	**%**
*Gender*		
Male	106	88
Female	25	12
		
*Age (years)*		
Median	60	
Range	37–78	
		
*Performance status (ECOG)*		
0	67	51
1	52	40
2	12	9
		
*Stage*		
IIIB (wet)	35	27
IV	96	73
		
*Histology*		
Adenocarcinoma	68	52
Squamous	56	43
Other	7	5
		
*Regimen*		
Docetaxel cisplatin	64	49
Docetaxel Gemcitabine	67	51
		
Response rate (CR+PR)	40	30
PFS (months, 95% CI)	4.2 (2.7–5.7)	
Median OS (months, 95% CI)	11.1 (9.7–14.6)	

Abbreviations: CR=complete response; PR=partial response; PFS=progression-free survival; OS=overall survival; CI=confidence interval; ECOG=Eastern Cooperative Oncology Group; DG=docetaxel and gemcitabine; DC=docetaxel and cisplatin.

**Table 1b:** *BRCA1*, *TXR1* and *TSP1* tumoural mRNA expression

	**All patients**	**Squamous**	**Non-squamous**	***P-*value** a
No. of patients (%)	131 (100)	56 (43)	75 (52)	
				
*BRCA1*				
Expression value, median (range)	4.28 (0.86–42.45)	8.1 (1.73–42.45)	3.62 (0.72–39.31)	0.001
High expression	65 (50)	28 (50)	37 (50)	
Low expression	66 (50)	28 (50)	38 (50)	
				
*TXR1*				
Expression value, median (range)	1.21 (0.02–7.8)	1.19 (0.02–6.7)	1.21 (0.12–7.8)	0.92
High expression	65 (50)	28(50)	36 (50)	
Low expression	66 (50)	28(50)	38 (50)	
				
*TSP1*				
Expression value, median (range)	0.24 (0.02–1.87)	0.23 (0.02–1.54)	0.24 (0.02–1.87)	1.0
High expression	65 (50)	28 (50)	36 (50)	
Low expression	66 (50)	28 (50)	38 (50)	

Abbreviations: *BRCA1*=*breast cancer 1 gene*; *TSP1*=*thrombospondin 1*; *TXR1*=*taxol resistance gene 1*.

a Mann–Whitney *U*-test, *P-*value.

**Table 2:** Tumoural expression of *BRCA1*, *TXR 1* and *TSP1* mRNA and treatment efficacy

		**PFS (months)**		**OS (months)**		**RR, *N* (%)**		
**Genes**	**No. of patients**	**Median (95% CI)**	***P-*value** a	**Median (95% CI)**	***P-*value** a	**CR+PR (%)**	**SD+PD(%)**	***P-*value**
*BRCA1* low	66 (50)	3.0 (2.3–3.7)	0.021	10.5 (6.2–14.8)	0.4	20	80	0.028
*BRCA1* high	65 (50)	6.0 (3.5–8.5)		11.2 (7.2–15.3)		42	58	
*TXR1* low	66 (50)	5.5 (2.1–8.9)	0.029	19.1 (10.4–27.9)	0.003	45	55	0.018
*TXR1* high	65 (50)	3.0 (1.8–4.0)		10.0 (7.4–12.7)		18	82	
*TSP1* low	66 (50)	2.6 (2.0–3.3)	<0.001	8.4 (5.2–11.6)	<0.001	22	78	0.035
*TSP1* high	65 (50)	6.1 (4.4–7.7)		25.1 (11.1–39.2)		41	59	

Abbreviations: *BRCA1*=*breast cancer 1 gene*; CR=complete response; RR=response rate; PR=partial response; PFS=progression-free survival; OS=overall survival; CI=confidence interval; SD=stable disease; PD=progressive disease; *TSP1*=*thrombospondin 1*; *TXR1*=*taxol resistance gene 1*.

a Log-rank *P-*value.

**Table 3:** Correlation of tumoural expression of *BRCA1*, *TXR1* and *TSP1* mRNA and treatment efficacy (a) in different histological subtypes and (b) across taxane-based regimens

			**PFS (months)**			**OS (months)**		
	**Genes**	**No. of patients**	**Median (95% CI)**	**Log-rank *P*-value**	**Interaction *P*-value**	**Median (95% CI)**	**Log-rank *P* -value**	**Interaction *P*-value**
(A)								
*Histology*								
Squamous	*BRCA1* low	28 (50)	4.0 (1.5–7.6)	0.047	0.61	11.3 (5.5–17.2)	0.99	0.97
	*BRCA1* high	28 (50)	7.4 (4.7–10.1)			9.8 (4.8–17.0)		
Non-squamous	*BRCA1* low	38 (51)	3.8 (2.1–5.5)	0.041		11.2 (8.3–16.1)	0.91	
	*BRCA1* high	37 (49)	7.2 (4.3–9.1)			11.1 (8.8–15.6)		
Squamous	*TXR1* low	28 (50)	7.1 (3.4–9.7)	0.038	0.21	16.4 (10.9–19.5)	0.022	0.19
	*TXR1* high	28 (50)	4.2 (1.6–7.8)			8.3 (2.6–12.1)		
Non-squamous	*TXR1* low	38 (51)	6.4 (3.8–9.6)	0.027		23.2 (10.2–38.1)	0.007	
	*TXR1* high	37 (49)	3.3 (1.9–3.6)			8.7 (5.2–12.3)		
Squamous	*TSP1* low	28 (50)	3.6 (1.4–7.3)	0.019	0.34	9.0 (4.6–13.5)	0.001	1.0
	*TSP1* high	28 (50)	6.1 (3.8–10.6)			25.2 (10.2–47.8)		
Non-squamous	*TSP1* low	38 (51)	2.3 (2.1–2.5)	0.001		7.9 (3.0–12.7)	0.001	
	*TSP1* high	37 (49)	6.3 (3.2–10.3)			23.2 (10.4–41.8)		
								
(B)								
*Regimen*								
Docetaxel–gemcitabine	*BRCA1* low	34 (51)	3.5 (2.1–4.8)	0.013	0.006	11.1 (6.8–15.5)	0.458	0.613
	*BRCA1* high	33 (49)	6.2 (4.1–8.5)			10.2 (3.9–17.6)		
Docitaxel–cisplatin	*BRCA1* low	32 (50)	6.8 (3.7–10.0)	0.874		11.2 (8.3–16.1)	0.912	
	*BRCA1* high	32 (50)	6.1 (4.5–7.7)			11.1 (8.8–15.6)		
Docetaxel–gemcitabine	*TXR1* low	34 (51)	7.2 (4.9–9.7)	0.024	0.56	15.8 (8.2–25.9)	0.013	0.387
	*TXR1* high	33 (49)	3.3 (2.1–4.4)			8.5 (4.1–12.8)		
Docitaxel–cisplatin	*TXR1* low	32 (50)	7.4 (4.2–10.3)	0.032		23.2 (10.2–38.1)	0.007	
	*TXR1* high	32 (50)	3.7 (1.4–5.2)			8.7 (3.9–12.3)		
Docetaxel–gemcitabine	*TSP1* low	34 (51)	3.4 (2.1–4.8)	0.007	0.48	7.5 (5.4–11.4)	0.006	0.931
	*TSP1* high	33 (49)	6.8(4.6–9.7)			26.7 (10.9–43.6)		
Docitaxel–cisplatin	*TSP1* low	32 (50)	4.4 (1.6–8.1)	0.002		9.1 (5.4–12.7)	0.002	
	*TSP1* high	32 (50)	7.6 (4.3–12.8)			36.4 (16.7–50.1)		

Abbreviations: *BRCA1*=*breast cancer 1 gene*; PFS=progression-free survival; OS=overall survival; CI=confidence interval; *TSP1*=*thrombospondin 1*; *TXR1*=*taxol resistance gene 1*.

**Table 4a:** Univariate analysis for PFS and OS

	**HR**	**95% CI**	***P*-value**
*PFS*			
*BRCA1* expression (high *vs* low)	**0.65**	**0.45–0.94**	**0.02**
*TXR1* expression (high *vs* low)	**1.61**	**1.17–2.43**	**0.002**
*TSP1* expression (high *vs* low)	**0.49**	**0.34–0.72**	**<0.001**
*TXR1*–*TSP1* expression (*TXR1* low/*TSP1* high *vs* others)	**0.60**	**0.39–0.90**	**0.01**
PS (2 *vs* 0–1)	1.66	0.89–3.10	0.13
Age (>70 years *vs* ⩽70 years)	1.17	0.77–1.84	0.48
Gender (male *vs* female)	1.30	0.72–2.33	0.38
Stage (IV *vs* III*β*)	**1.90**	**1.15–2.58**	**0.02**
			
*OS*			
*BRCA1* expression (high *vs* low)	1.02	0.69–1.50	0.93
*TXR1* expression (high *vs* low)	**1.83**	**1.23–2.73**	**0.003**
*TSP1* expression (high *vs* low)	**0.34**	**0.22–0.53**	**<0.001**
*TXR1*–*TSP1* expression (*TXR1* low/*TSP1* high *vs* others)	**0.38**	**0.24–0.62**	**<0.001**
PS (2 *vs* 0–1)	**1.92**	**1.02–3.58**	**0.04**
Age (>70 years *vs* ⩽70 years)	1.27	0.79–2.04	0.33
Gender (male *vs* female)	1.57	0.77–3.59	0.18
Stage (IV *vs* III*β*)	**1.73**	**1.03–2.24**	**0.04**

Abbreviations: *BRCA1*=*breast cancer 1 gene*; PFS=progression-free survival; PS=performance status; OS=overall survival; CI=confidence interval; HR=hazard ratio; *TSP1*=*thrombospondin 1*; *TXR1*=*taxol resistance gene 1*. Values shown in bold are statistically significant.

**Table 4b:** Multivariate analysis for time to tumour progression and OS

	**HR**	**95% CI**	***P*-value**
*PFS*			
Stage (IV *vs* III*β*)	1.41	0.94–1.66	0.09
*BRCA1* expression (high *vs* low)	**0.53**	**0.37–0.78**	**0.001**
*TXR1*–*TSP1* expression (*TXR1* low/*TSP1* high *vs* others)	**0.49**	**0.32–0.76**	**<0.001**
			
*OS*			
Stage (IV *vs* III*β*)	1.42	0.92–2.74	0.2
*TXR1*–*TSP1* expression (*TXR1* low/*TSP1* high *vs* others)	**0.37**	**0.23–0.58**	**<0.001**
PS (0–1 *vs* 2)	**1.92**	**1.02–3.60**	**0.04**

Abbreviations: *BRCA1*=*breast cancer 1 gene*; PFS=progression-free survival; PS=performance status; OS=overall survival; CI=confidence interval; HR=hazard ratio; *TSP1*=*thrombospondin 1*; *TXR1*=*taxol resistance gene 1*. Values shown in bold are statistically significant.
