# Supplementary material for: Correlation of BRCA1, TXR1 and TSP1 mRNA expression with treatment outcome to docetaxel-based first-line chemotherapy in patients with advanced/metastatic non-small-cell lung cancer
Source: Br J Cancer. 2010 Dec 14;104(2):316–23. doi: 10.1038/sj.bjc.6606027 (PMC3031890; doi:10.1038/sj.bjc.6606027)
Supplement: Supplementary Figures [file 6606027x1.doc]

**Supplementary Figure 1a**


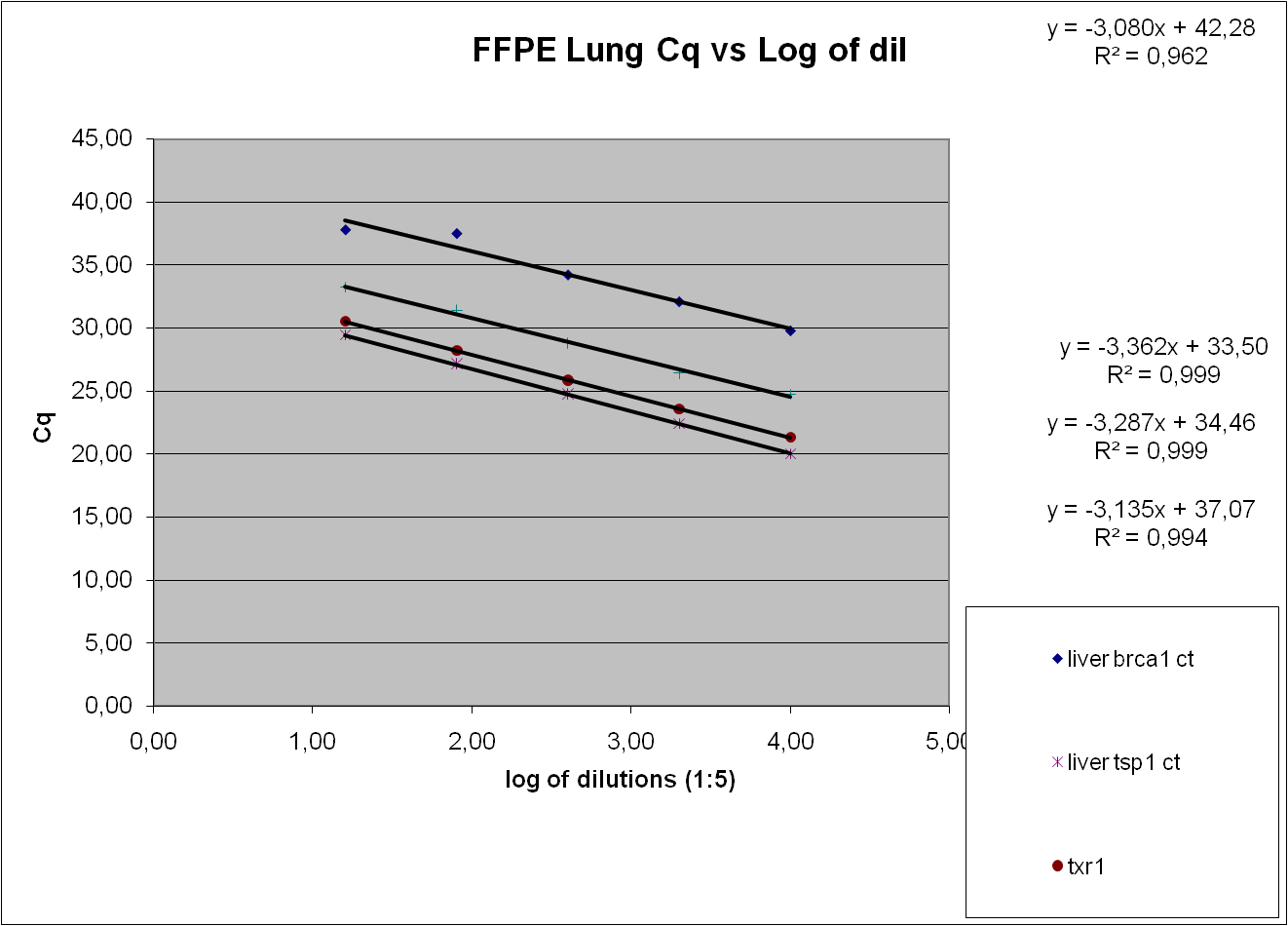


**Supplementary Figure 1b**


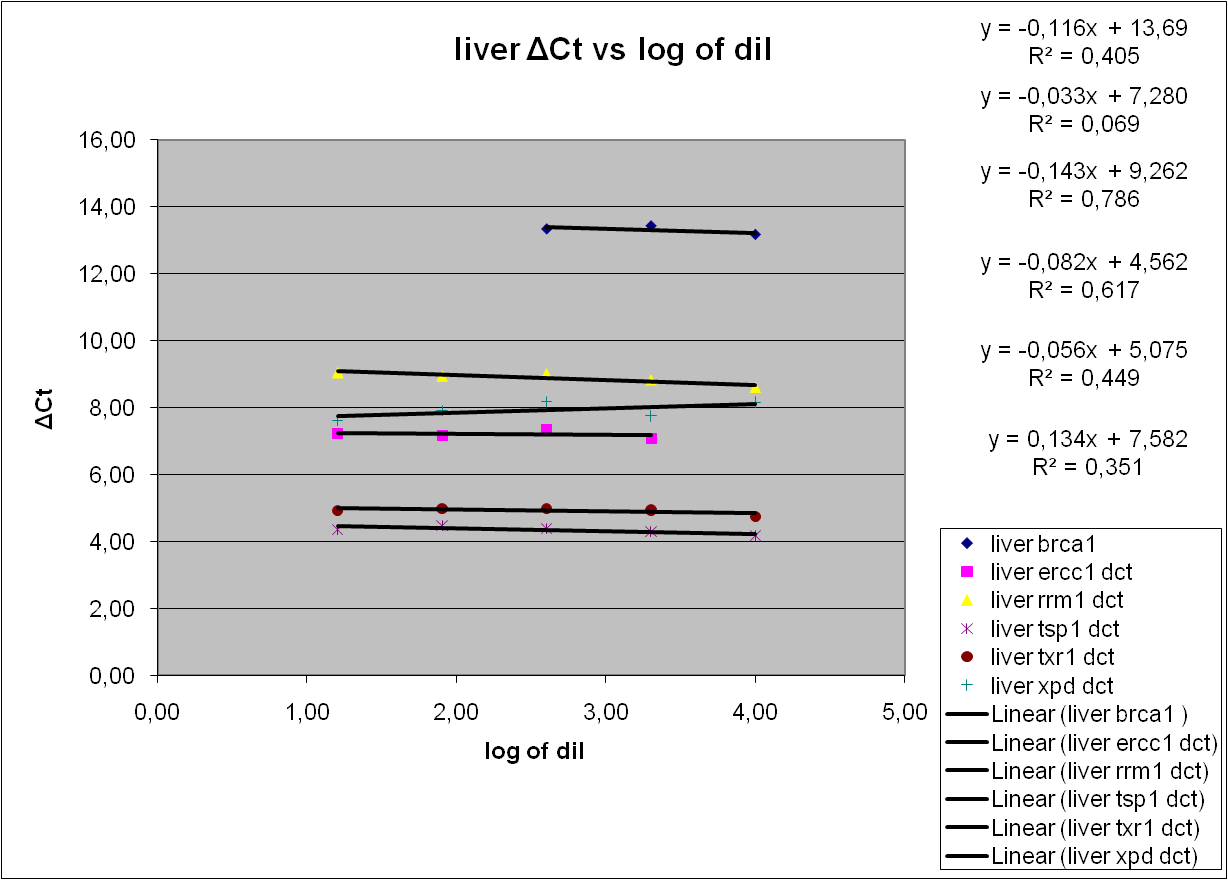


**Supplementary Figure 2a**

**
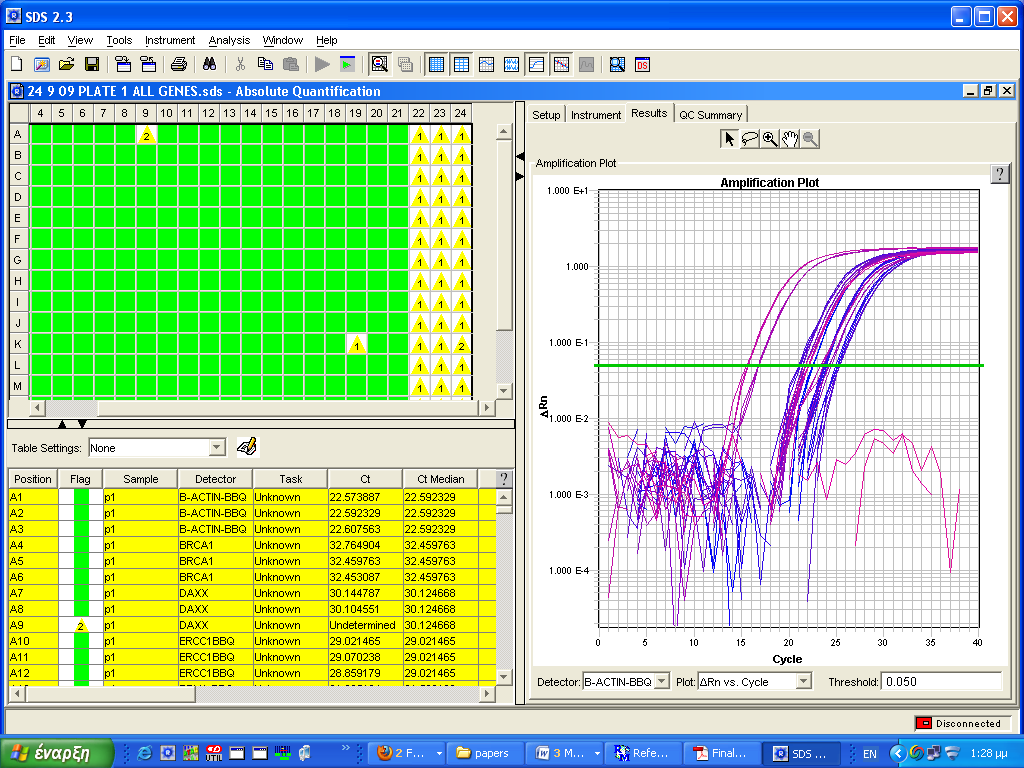
**

**Supplementary Figure 2b**

**
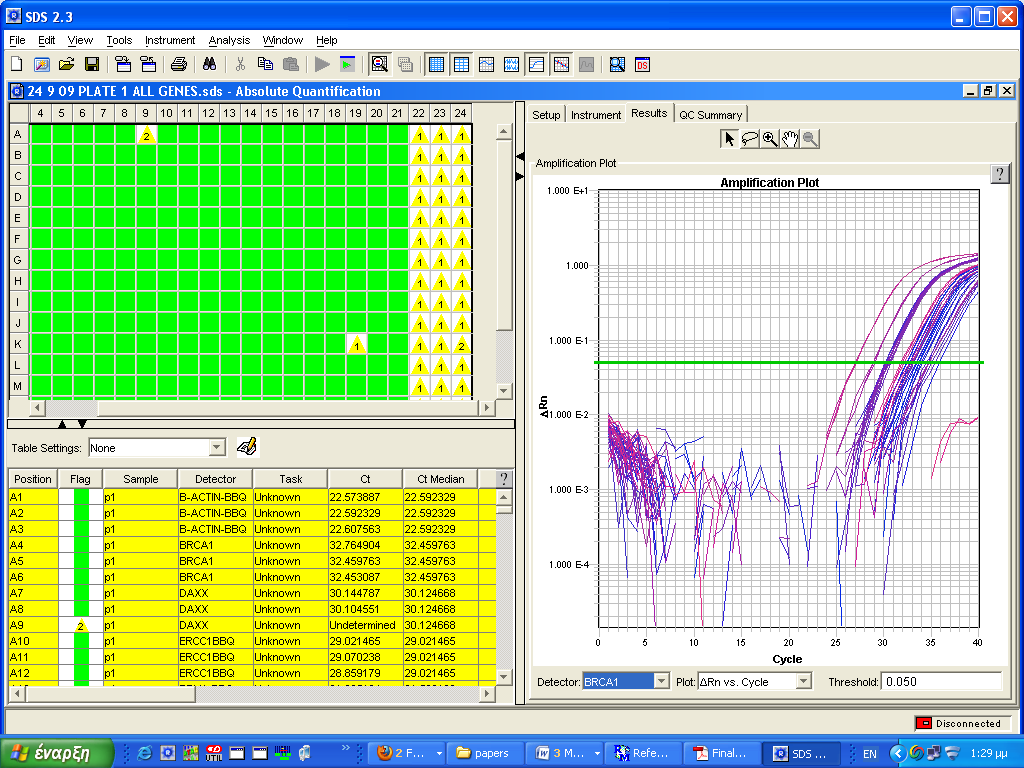
**

**Supplementary Figure 2c**

**
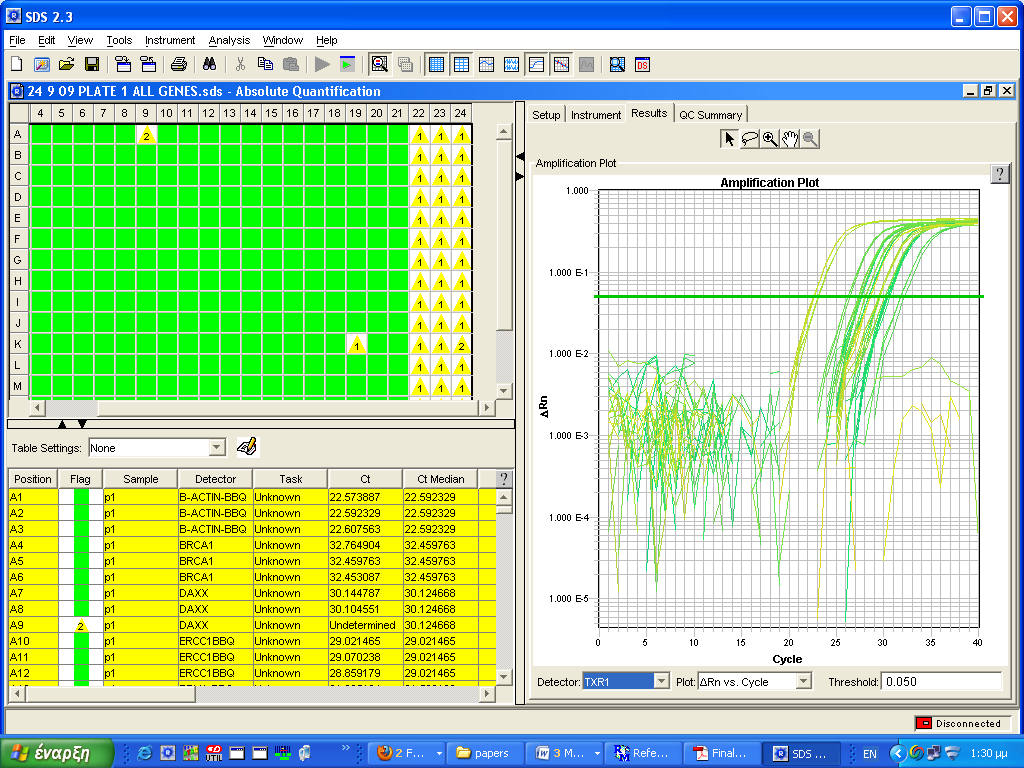
**

**Supplementary Figure 2d**

**
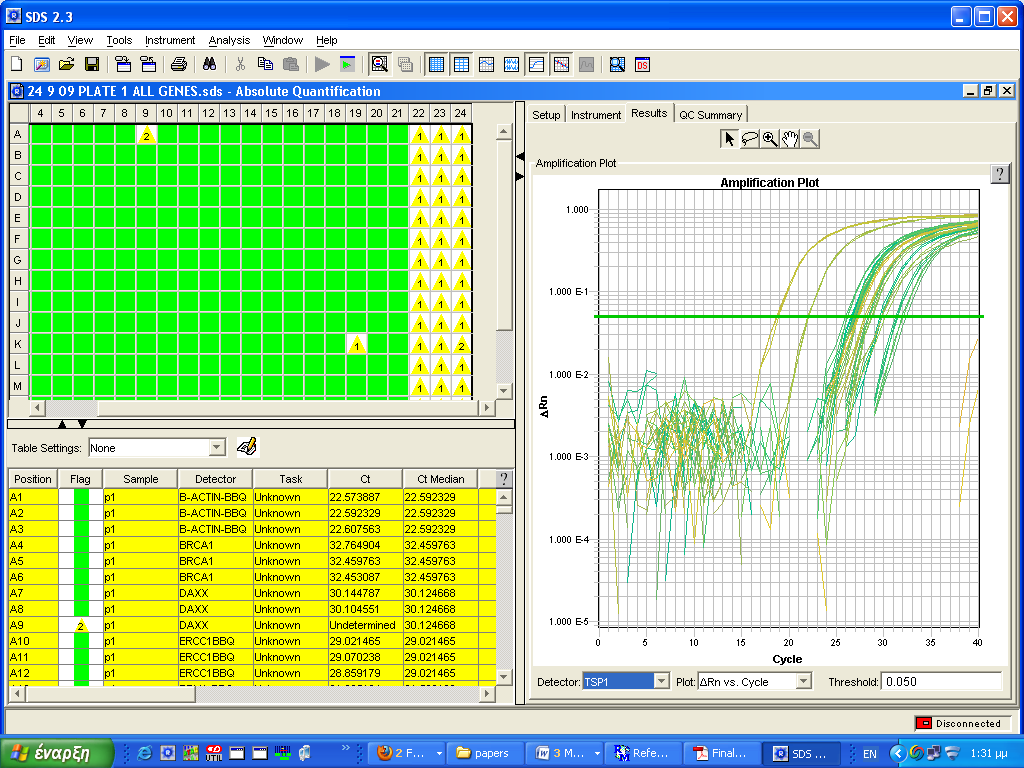
**
